# Nanostructured Polyacrylamide Hydrogels with Improved Mechanical Properties and Antimicrobial Behavior

**DOI:** 10.3390/polym14122320

**Published:** 2022-06-08

**Authors:** Elena Olăreț, Ștefan Ioan Voicu, Ruxandra Oprea, Florin Miculescu, Livia Butac, Izabela-Cristina Stancu, Andrada Serafim

**Affiliations:** 1 Advanced Polymer Materials Group, University Politehnica of Bucharest, 011061 Bucharest, Romania; elena.olaret@upb.ro (E.O.); stefan.voicu@upb.ro (Ș.I.V.); izabela.stancu@upb.ro (I.-C.S.); 2 Faculty of Chemical Engineering and Biotechnologies, University Politehnica of Bucharest, 011061 Bucharest, Romania; ruxandra_aela@yahoo.com (R.O.); livia_butac@hotmail.com (L.B.); 3 Faculty of Materials Science and Engineering, University Politehnica of Bucharest, 060042 Bucharest, Romania; florin.miculescu@upb.ro

**Keywords:** nanostructured polyacrylamide, silver-decorated carbon nanotubes, mechanical properties, antibacterial activity

## Abstract

This work proposes a simple method to obtain nanostructured hydrogels with improved mechanical characteristics and relevant antibacterial behavior for applications in articular cartilage regeneration and repair. Low amounts of silver-decorated carbon-nanotubes (Ag@CNTs) were used as reinforcing agents of the semi-interpenetrating polymer network, consisting of linear polyacrylamide (PAAm) embedded in a PAAm-methylene-bis-acrylamide (MBA) hydrogel. The rational design of the materials considered a specific purpose for each employed species: (1) the classical PAAm-MBA network provides the backbone of the materials; (2) the linear PAAm (i) aids the dispersion of the nanospecies, ensuring the systems’ homogeneity and (ii) enhances the mechanical properties of the materials with regard to resilience at repeated compressions and ultimate compression stress, as shown by the specific mechanical tests; and (3) the Ag@CNTs (i) reinforce the materials, making them more robust, and (ii) imprint antimicrobial characteristics on the obtained scaffolds. The tests also showed that the obtained materials are stable, exhibiting little degradation after 4 weeks of incubation in phosphate-buffered saline. Furthermore, as revealed by micro-computed tomography, the morphometric features of the scaffolds are adequate for applications in the field of articular tissue regeneration and repair.

## 1. Introduction

Hydrogels are used for the fabrication of scaffolds with biomedical applications due to their ability to uptake large amounts of water and their resemblance to the extracellular matrix, but, usually, their low mechanical resistance to effort limits their use as tissue substitutes. To address this issue, several approaches have been proposed, including the synthesis of interpenetrated networks (IPNs) [1,2], reinforcement with micro- or nanospecies [3], or the use of specific combinations of two or more components [4]. Owing its broad usefulness to properties such as chemical inertia, tailorable mechanical properties, high swelling degree, and optical transparency, polyacrylamide (PAAm) has been intensively investigated for various applications in the biomedical field, ranging from the fabrication of contact lenses [5] to bone tissue engineering [6,7]. Although they exhibit adjustable elasticity, PAAm hydrogels are usually purely elastic, displaying little or no dissipation of deformation energy, unlike natural soft tissue, which is viscoelastic [8]. Recent studies have exploited routes of obtaining viscoelastic PAAm hydrogels, with mechanical properties, which show a closer resemblance to native soft tissue [8,9]. A more usual approach to modifying the mechanical properties of hydrogels is represented by nanostructuring. As a natural consequence of using fillers in a polymeric matrix, the mechanical properties are also altered, usually leading to more mechanically robust material.

Nanostructuring is being increasingly researched in various domains, including the development of new materials with biomedical applications. In this respect, carbon nanostructures have gained a lot of attention due to their mechanical, electronic, and biological properties [10]. Available in various structural forms—0D (fullerenes, carbon dots), 1D (single-wall carbon nanotubes (SWCNT) or multi-wall carbon nanotubes (MWCNT)), 2D (graphene), or 3D (nanodiamonds)—carbon nanostructures offer promising potential for the development of several types of materials, with medical applications ranging from biosensors [11,12,13] to drug delivery systems [14,15,16] or structures for tissue engineering [17,18,19]. Among the investigated nanospecies, carbon nanotubes (CNTs) represent an appealing candidate, due to their high conductivity, high strength, biocompatibility and ease of functionalization [20]. However, as most nanospecies, their use is restricted, due to their low dispersibility, especially in aqueous media [21]. Nonetheless, the presence of functional groups [22,23] or appropriate ultrasonication treatment [24,25,26] has led to improved results and ultimately to materials with superior characteristics. The viscosity of the polymeric matrix [27] plays an important role in obtaining a homogeneous distribution of the nanospecies in a CNT-based composite. In highly viscous matrices, the mobility of the nanospecies is restricted, thus preventing the agglomeration and sedimentation of the nanoscale filler.

Moreover, several research studies have demonstrated that CNTs also possess strong inhibitory effects against both Gram-negative and Gram-positive microorganisms when placed in direct contact, either through interactions in aqueous solutions [28] or as surface coatings [29,30]. CNTs inhibit the bacterial growth, either due to their geometry and small dimensions, which permit the breakage of the cellular membrane resulting in cell’s death [31], or by disrupting the equilibrium between the production of free radicals and antioxidant defenses, thus resulting in oxidative stress followed by cell death [32,33]. Due to their notable properties, CNTs’ compatibility with the live tissue has been scrutinized, and intensive research has been performed to this end [34]. Due to their small dimensions and structural similarity to asbestos, CNT inhalation has been investigated, and their effect on the respiratory tract has been documented as by some studies as harmful [35,36,37,38]. There are studies that suggest that CNTs’ cytotoxicity and overall effect on cells are strongly influenced by a series of factors, such as CNT type, surface chemistry and concentration, and exposure time [39]. However, when used in the fabrication of composites for tissue engineering, CNTs’ behavior differs considerably from that of inhaled CNTs [40]. When embedded in a biocompatible matrix, the toxicity of these nanospecies is hampered, but they are able to provide composites with remarkable structural reinforcement or electro-conductive properties, which are adequate for bone [41] or nerve regeneration and repair [42], respectively. Furthermore, incorporating CNTs into polymeric matrices imprints a certain degree of antimicrobial activity on the material, depending on the ratio, type, and features of the used nanospecies, treatment time, and the characteristics of the microorganism [30,43,44]. To further increase the antibacterial activity of CNTs, various molecules are adsorbed on their surface (e.g., silver [45,46], peptides [47], enzymes [48], etc.), and the so-obtained decorated CNTs were further used to obtain composites with the ability to inhibit the activity of various microorganisms. The design of materials that, in addition to being able to repair or replace a certain tissue, also possess antimicrobial characteristics [49] or release in a controlled manner certain active species [50] is of great interest to material scientists, and extensive efforts have been made in this direction.

The present study proposes a one-pot synthesis of nanostructured viscoelastic materials with applications in the low-load bearing articular tissue regeneration and repair. Additionally, this study shows that the incorporation into the monomer-cross-linker system of the corresponding linear polymer leads to an elastic, high-performance material that is able to withstand repeated compressions. The microarchitectural features of lyophilized materials were investigated through micro-computed tomography, and the morphometric parameters were correlated with the samples’ composition. The stability of the samples was investigated through the incubation in acellular media for 28 days. Furthermore, this work also investigates the antimicrobial behavior of the composites when various ratios of silver decorated CNTs are used for nanostructuring.

## 2. Materials and Methods

The monomer (acrylamide, AAm), crosslinker (*N,N*-Methylenebis(acrylamide), MBA), and carbon nanospecies (carboxyl-functionalized multiwall carbon nanotubes, CNTs) were purchased from Sigma. Polyacrylamide (PAAm), with an average molecular weight of 3.76 × 10^5^ g/mol, was obtained in the laboratory through the free radical photopolymerization of the monomer in an aqueous solution in the presence of Irgacure 2959 (Sigma, St. Louis, MO, USA), purified through dialysis against distilled water (dH_2_O) using dialysis membranes (SpectraPor, MWCO 12–14 kDa, Repligen, CA, USA). The polymer’s synthesis and the method employed for the assessment of its viscosity and average molecular weight are described in Appendix A. Ammonium persulphate (APS, Sigma) and trietanolamine (TEA, Sigma) were used as the redox initiating system of the AAm-MBA system. Phosphate-buffered saline (PBS 0.01 M, pH 7.4, Sigma-Aldrich, St. Louis, MO, USA) was prepared according to manufacturer’s instructions. For the antimicrobial tests, *Staphylococcus aureus* (ATCC 6538TM) and *Escherichia coli* (ATCC 10536TM) prepared according to the manufacturer’s recommendations were used as references. Casein soyabean digest broth (TSB, Sigma) was used as dispersant for the lyophilized bacteria, while casein soyabean digest agar (TSA, Sigma) was used as culture medium.

### 2.1. Synthesis

Two series of samples were synthesized to highlight the effect of embedding linear PAAm in the classical 3D polymeric network of PAAm-MBA. For simplicity, the two series were further denominated AC (acrylamide-carbon nanotubes) and PAC (polyacrylamide-acrylamide-carbon nanotubes). The two series were prepared following the same protocol, as described below for the AC series.

Firstly, various amounts of CNTs were ultrasonicated in dH_2_O for 90 min, as described in Table 1. The adequate amounts of monomer (acrylamide—AAm, 10% wt./vol. in the final solution) and crosslinker (*N,N*-Methylenebis(acrylamide)—MBA, AAm:MBA = 100:2 wt./wt.) were added under vigorous stirring at room temperature. After the complete dissolution of all reagents, the initiators—ammonium persulfate (APS) and triethanolamine (TEA)—were added, and the hydrogel precursor was poured into molds. After two hours at 37 °C, all hydrogels (except the samples used for establishing the polymerization efficiency) were removed from the molds and washed with large amounts of dH_2_O for 24 h at 40 °C under gentle stirring. In the case of PAC series, the PAAm was added in the polymerization mixture maintaining a ratio of AAm:PAAm = 100:25 (wt./wt.) immediately after the dissolution of the AAm and MBA.

The PAC series was further used to establish the minimum ratio of silver that would imprint a relevant antimicrobial effect. To this end, the same synthesis protocol was followed, but the CNTs were decorated with silver ions (Ag) though the direct dispersion of the nanospecies in AgNO_3_ aqueous solution, maintaining a CNT:Ag ratio of 100:1 (wt./wt.). For simplicity, this series will be denominated Ag@PAC below.

### 2.2. Polymerization Efficiency

The polymerization efficiency was assessed though gel fraction (*GF*, %), as described in [2]. Immediately after synthesis, samples of each composition were dried in the oven at 37 °C for 24 h and weighted (*w_i_*). Then, they were thoroughly washed with dH_2_O for 48 h, at 37 °C under mild stirring, dried, and weighted again (*w_f_*). *GF* was determined using Equation (1):

(1) $$GF,\;\%=\frac{w_{f}-w_{i}}{w_{i}}\times 100$$

where *w_i_* is the initial mass of the dried samples as they result from the crosslinking process and *w_f_* is the mass of the dried samples after extraction in dH_2_O at 37 °C for 48 h.

### 2.3. Water Uptake Ability

The swelling ability of the hydrogels was assessed following the protocol described in [51]. Briefly, samples with a diameter of 6 ± 0.2 mm and a height of 10 ± 0.5 mm were incubated in plastic tubes containing 15 mL of dH_2_O and placed in the oven for 24 h, at 37 °C. The hydration equilibrium was considered achieved when three identical consecutive measurements performed at relevant timepoints (2 h) were obtained. The tests were performed in triplicate for each composition. The swelling degree (*SD*, %) was determined gravimetrically using Equation (2):

(2) $$SD,\;\%=\frac{w_{f}-w_{0}}{w_{0}}\times 100$$

where *w_o_* denotes the initial mass of the samples prior to incubation and *w_f_* is the mass of the equilibrium-hydrated samples. Before the test, the samples were purified in large amounts of water for 24 h and subsequently dried in the oven, at 37 °C, for 24 h.

### 2.4. Investigation of the Morphometric Parameters

The morpho-structural characteristics of the synthesized hydrogels were assessed using micro-computed tomography (micro-CT) performed on lyophilized samples. To this end, samples of both series were hydrated at equilibrium and subsequently lyophilized using Martin Christ equipment (−80 °C, 48 h, under vacuum). After freeze drying, the materials were kept at room temperature in sealed containers to avoid humidity. No changes in the shape and dimensions of the samples were noticed during storage. A SkyScan 1272 high-resolution X-Ray micro-tomograph (Bruker MicroCT, Belgium) was used to assess the morphometric parameters of the porous materials. The projections were recorded at a camera binning of 1 × 1 with a voltage of 50 kV and an emission current of 130 µA. All samples were scanned at a pixel size of 1.25 μm with 4 average frames at every 0.1° angle step. The projections were reconstructed using the NRecon software (version 1.7.1.6, Bruker, Kontich, Belgium), and the obtained cross-sections were further used to obtain 3D images of the scanned samples using CTVox software (version 3.3.or1403, Bruker, Kontich, Belgium). The porosity measurements were performed maintaining the same volume of interest for all samples, using the CTAnalyzer software (version 1.18.4.0+, Bruker, Kontich, Belgium).

### 2.5. Mechanical Properties

The mechanical characteristics of AC and PAC series were assessed through uniaxial compression tests performed using a CT3 Texture analyzer (Brookfield Engineering Laboratories Inc., Middleboro, MA, USA) equipped with a 4500 g cell load and a TA4/1000 compression accessory. Cylindrical samples of each composition (d = 10 ± 0.5 mm, h = 6 ± 0.2 mm), hydrated to equilibrium, were placed on the bottom plate of the equipment, and the upper plate was lowered up to a certain deformation. Ten cyclic loading-unloading tests were performed up to a deformation of 50% on the same sample immediately after the initial loading at a crosshead speed of 0.1 mm/s to determine the elasticity and deformation of the materials during subsequent exposure to effort. The stress–strain curve was plotted using the dedicated software (TexturePro CT V1.8 Build 31). The stress was read in the linear region at 2% deformation on the loading curve of the first cycle and the compression modulus (*E*′, kPa) was computed using Equation (3). Hysteresis was also computed using Equation (4) at 40% deformation in the first and last loading–unloading cycle. Single loading tests were performed with a crosshead speed of 1 mm/s to determine the ultimate compression stress the material can withstand before breakage.

(3) $$E^{\prime },\;kPa=\frac{\sigma }{\varepsilon }=\frac{\frac{F}{A}}{\frac{\Delta L}{L}}$$

where

*E*′ = compression modulus, (kPa)
σ = applied compressive stress, (kPa)
ε = strain
*F* = applied compressive force, (kN)
*A* = samples’ area, (m^2^)
ΔL = compressed length, (m)
*L* = original length of the sample, (m)

(4) $$H,\;\%=\frac{\sigma_{up}-\sigma_{d}}{\sigma_{max}}\times 100$$

where

*H* = hysteresis, (%)
σ*_up_* = the value of stress read on the loading curve, (kPa)
σ*_d_* = the value of stress read on the unloading curve, (kPa)
σ*_max_* = the maximum value of stress, (kPa)

### 2.6. Stability in Acellular Medium

Hydrogels’ stability was investigated in acellular conditions using as incubation medium phosphate-buffered saline (PBS), as described in [52]. Briefly, three samples of each composition (d = 10 ± 0.5 mm, h = 6 ± 0.2 mm) were kept for 4 weeks in individual test tubes containing 15 mL of PBS, at a constant temperature (37 °C) on an orbital rotator (IKA KS 4000) at 50 rpm. The incubation medium was refreshed every two days throughout the experiment. The remaining mass (*RM*, %) was calculated using Equation (5):

(5) $$RM,\;\%=\frac{w_{f}}{w_{i}}\times 100$$

where *w_i_* is the weight of dried specimen before incubation in PBS and *w_f_* is the weight of dried specimen after incubation.

### 2.7. Antimicrobial Activity

The antibacterial activity of the compositions was determined through indirect contact, as described in [53]. Briefly, bacterial suspensions of *E. coli* and *S. aureus* (concentration of colonies of 10^3^ CFU/mL) were placed in contact with samples of each composition. The positive control was evaluated by bacterial suspension incubation in polypropylene sterile tubes, in the same conditions as analyzed samples (without contact with any other material). Following 24 h of incubation at 30–35 °C, PBS was added over the samples, and the aliquot was transferred to Petri dishes and spread onto fresh agar plates to acquire images and count the colonies. The samples were incubated for 3 days at 30–35 °C. The antimicrobial activity was computed using Equation (6):

(6) $$AA,\;\%=\frac{N_{i}-N_{c}}{N_{i}}\times 100$$

where

*AA* represents the antibacterial activity;
*N_i_* represent the initial number of bacterial colonies (1000 CFU/mL);
*N_c_* represents the obtained number of bacterial colonies.

### 2.8. Statistical Analyses

All experiments were conducted in triplicate (*n* = 3), and results are expressed as mean ± standard deviation. Statistical relevance was performed using GraphPad Prism Software 6.0 (GraphPad Software Inc., San Diego, CA, USA), one-way ANOVA method, Bonferroni post-test, and differences were considered statistically significant for *p* < 0.05.

## 3. Results and Discussions

The present paper discusses the possibility of modulating the mechanical performances of PAAm hydrogels using two different approaches simultaneously: on one hand, the nanostructuring of the polymeric matrix with CNTs, and on the other hand, the addition of the corresponding linear polymer in the hydrogel precursor mixture. While the first route would lead to stiffer materials, it is expected that the second one would imprint a certain elasticity on the scaffolds. In addition to tailoring the mechanical properties of the classical MBA-cross-linked PAAm hydrogels through the addition of linear PAAm and CNTs nanostructuring, the study also aimed at establishing the lowest Ag:CNTs ratio that imprints a relevant antimicrobial behavior on the obtained scaffolds.

**Figure 1:**
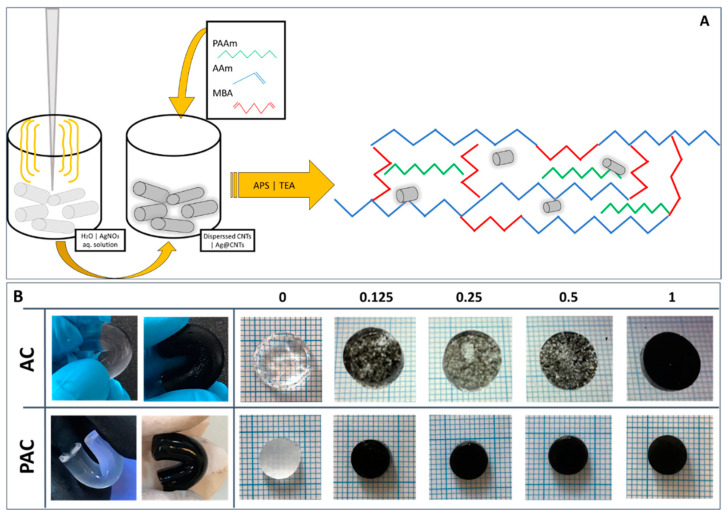
Panel (**A**): schematical depiction of the synthesis process of the polyacrylamide-based hydrogels reinforced with Ag-decorated CNTs; Panel (**B**): digital images of the obtained hydrogels at hydration equilibrium.

The one-pot synthesis of the materials consisted of two preparatory steps: (1) first, the CNTs dispersion/Ag decoration, performed through the sonochemical method, followed by (2) the addition of the polymer matrix components (monomer, cross-linker, and linear polymer) and polymerization initiators (Figure 1A). For the Ag@PAC series, during the first step, the cavitation bubbles generated by the ultrasonical field enabled the decoration of the CNTs [54] with Ag ions from the AgNO_3_ aqueous solution. A notable aspect regarding the designed materials is represented by the low ratio of both CNTs in the system (a maximum of 1% weight ratio with respect to the monomer) and low ratio of metallic ions (only 1% Ag weight ratio with respect to the CNTs).

The resulting hydrogels are elastic and smooth. Figure 1, panel B, presents aggregates of CNTs observed in the majority of AC compositions. The addition of the linear polymer leads to hydrogels with a homogeneous appearance, which may be assigned to an improved dispersion of the CNTs in the polymeric matrix (PAC series in Figure 1B). These data support the hypothesis that PAAm acts as dispersion stabilizer.

PAAm was previously used to enhance the elasticity of PAAm-MBA hydrogels in order to better resemble natural tissues [8,9]. To the best of our knowledge, this paper is the first to report the employment of this synthesis method to obtain composite materials.

### 3.1. Polymerization Efficiency

**Figure 2:**
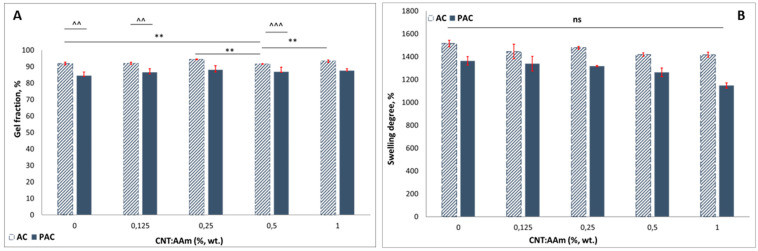
Gel fraction (**A**) and swelling degree (**B**) values computed for the AC (patterned columns) and PAC (full color columns) series. Statistical significance: ^^, ** *p* < 0.01, ^^^ *p* < 0.001, ns—not statistically significant.

The polymerization efficiency of the materials was determined through gel fraction analysis. Values above 84% were registered for the PAC series and above 90% for the AC compositions (Figure 2A). The values registered for PAC series are slightly lower than the ones registered for the classically synthesized AC series. This behavior may be attributed to the high content of linear polymer added in the system (25% with respect to the monomer), which was not completely trapped in the 3D network of the PAAm-MBA hydrogel. Considering that the linear PAAm amounts for about 20% (wt./wt.) of the total solid content of the polymerization mixture, the GF values confirm the presence of the linear polymer in the final purified material. Additional details regarding the stability of the purified scaffolds are presented in “Section 3.5. Stability in simulated physiologic conditions”.

### 3.2. Water Uptake Ability

The swelling in dH_2_O was assessed through the classical gravimetric method, as described in the methods section. Although at low CNT fillings (0.125 and 0.25% CNTs with respect to the AAm content) there are no notable differences between the neat hydrogel and the composites, at filling ratios above 0.5% CNTs, a slight decrease in the SD value can be observed in both AC and PAC series (Figure 2B). This behavior can be attributed to the presence of CNTs above a critical filling ratio; the presence of the CNTs in the network’s spaces obstructs water accumulation. For example, the SD value of AC-0 is 1515 ± 30%, while for AC-1, the SD reaches 1148 ± 23%. Furthermore, some differences might also be noticed between the SD values of the two series. The SD decrease in the PAC series when compared to the AC series can also be assigned to an increase in the total solid content in the composition. Although not statistically significant, the addition of CNTs impacts the PAC water affinity when compared to the AC series: SD of AC-1 is 6.5% lower than AC-0, while the SD of PAC-1 is almost 16% lower than the neat semi-IPN hydrogel. This behavior shows that the water absorption was additionally reduced by the presence of the CNTs in the PAC network.

### 3.3. Architectural Characteristics

**Figure 3:**
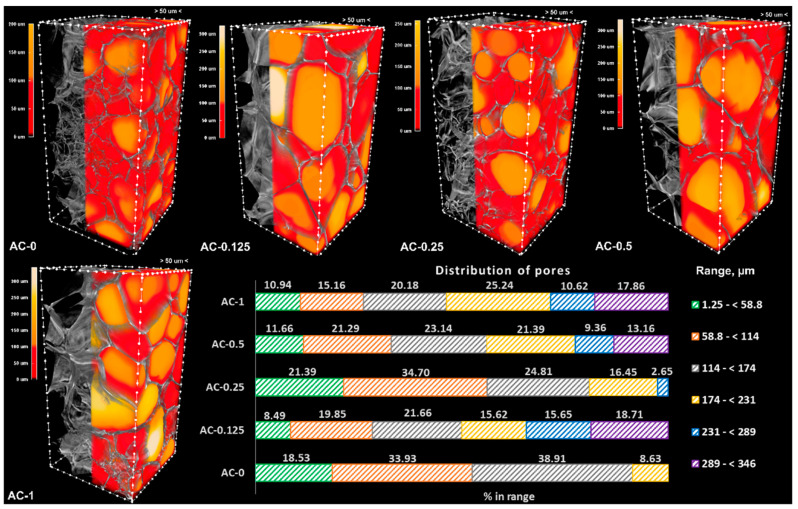
Three-dimensional images of the AC samples and graphical representation of the porosity measurements.

**Figure 4:**
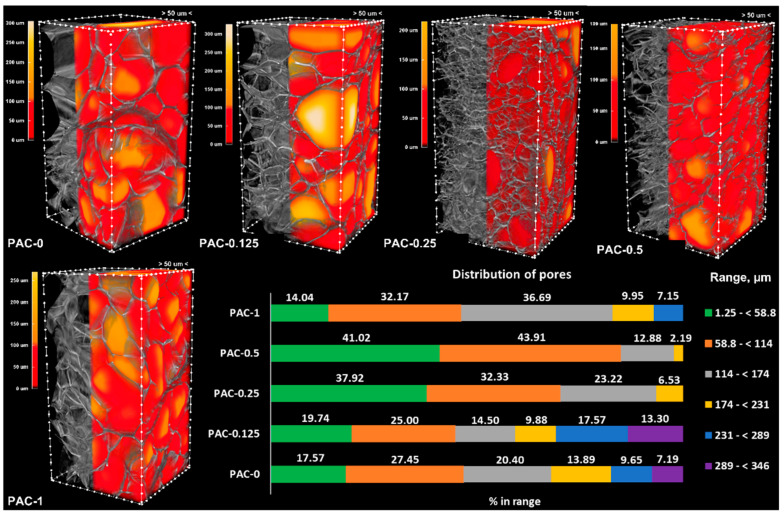
Three-dimensional images of the PAC samples and graphical representation of the porosity measurements.

The morphometric parameters of the hydrogels were assessed on porous samples resulting from freeze drying, using a micro-CT scanner at a resolution of 1.25 µm. The images were visualized as 3D objects, and the pores were color-coded yellow to red according to their dimensions, while the walls were kept on a grey scale. Structure separation was quantitatively evaluated, and the results were graphically represented as bar charts (Figure 3 and Figure 4). The morphometric parameters (Table 2) were obtained for the same volume of interest (VOI) and number of layers, maintaining the upper and lower grey threshold constant for all samples. The graphical representation presented in Figure 3 and Figure 4 shows the percentage of pores space within different range values in the VOI.

Both series have a total porosity of around 90%, consisting mostly of opened pores. The addition of the linear PAAm in the system leads to an overall decrease in porosity (for example, in the case of the neat hydrogels, the total porosity decreases from 92.6% for AC-0 to 89.1% for PAC-0), but the addition of the CNTs cannot be correlated with a clear trend of the morphometric parameters. However, the fact that the CNTs were added in low amounts must not be overlooked (the maximum amount of CNTs is 1% with respect to the monomer, the equivalent of 0.1% (wt.) in the final system). Moreover, in the case of the AC series, the results registered for the specific surface area could not be correlated with the composition, probably due to the poor distribution of the CNTs within the polymer matrix.

As depicted in Figure 3, the control sample (AC-0) is lacking pores greater than 231 µm, with most of them (over 52%) being in the range of 1.25–114 µm. The addition of nanoparticles in the system leads to an increase in the pores’ dimensions and to the appearance of larger ones, with dimensions in the interval 231–346 µm; the sample with the highest content of CNTs has the majority of pores (53.72%) in the interval 174–346 µm.

For the PAC series, the results indicated an opposite trend, with the neat hydrogel exhibiting larger pores when compared to the nanocomposites. This behavior indicates that the addition of the linear PAAm in the system has a greater influence when compared to the addition of CNTs, especially since the PAAm content is much higher (25-fold when compared to the maximum load of CNTs). As presented in Figure 4, the samples with the lowest pore dimensions are PAC-0.5 and PAC-0.25, which present only pores lower than 231 µm, with the vast majority (over 85% for PAC-0.5 and 70% for PAC-0.125) lower than 114 µm.

Materials designed for articular cartilage tissue regeneration and repair require open porosity, which is useful for scaffolds’ colonization with chondrocytes. These are specialized cells found in the articular cartilage with dimensions in the range of 7–30 µm [55]. However, studies have shown that increasing both porosity and pore size leads to improved cell viability [56,57]. As presented in Table 2, both series of materials exhibited great open porosity, but series PAC, especially compositions PAC-0.25 and PAC-0.5, have a large number of pores in the relevant porosity interval, considerably higher than their AC counterparts (patterned green for AC in Figure 3 and green for PAC in Figure 4).

The surface morphology of the samples was also investigated through scanning electron microscopy, using a FEI XL30 equipment. No noticeable differences were observed between series AC and PAC. The registered images showed that all samples have heterogeneously distributed pores, with smooth surfaces. Relevant images of the scaffolds are presented in Supplementary Figure S3.

### 3.4. Mechanical Behavior

**Figure 5:**
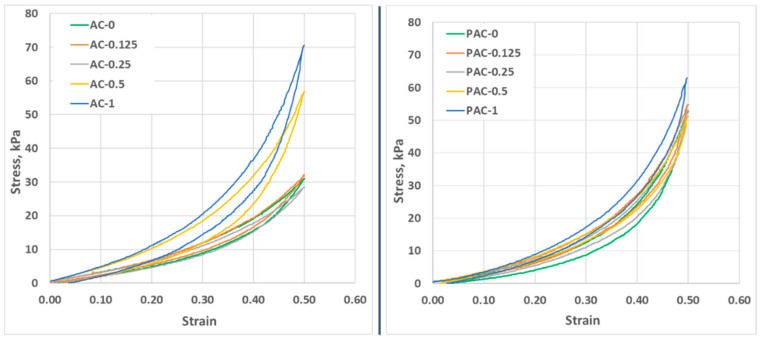
First cycle of loading–unloading compressions for the AC (**left**) and PAC (**right**) series.

The fatigue resistance of the materials was evaluated through cyclic loading–unloading tests performed in a quasi-static manner at a low compression speed (0.1 mm/s). As depicted in Figure 5, all samples exhibited good elasticity, as the stress decreased gradually after load removal. There were notable differences between the AC and PAC samples. Firstly, in the case of the AC series, hysteresis may be observed even at low strain values (strain 0.5), especially above a 0.5% filling ratio (wt., CNT:AAm), while in the case of PAC samples, hysteresis may be observed at slightly higher values. Even more, the hysteresis of the PAC samples is lower when compared to the values computed for the AC series (Table 3). In the first compression cycle, there are no notable differences between the hysteresis values of the neat hydrogels of the two series (11.55% for AC-0 and 11.37% for PAC). The hysteresis of the neat hydrogels is significantly altered by the addition of nanoparticles in both series. For the AC series, the hysteresis of the low-loading composites (AC-0.125 and AC-0.25) is smaller, while for the compositions with 0.5% and 1% filling ratios (AC-0.5 and AC-1, respectively), it is higher than the neat hydrogel. AC-0.5 and AC-1 behave quite differently under stress compared to the rest of the compositions of the same series, even at 10% deformation, as depicted in Figure 5. The plotted strain–stress curves show that when AC-0.5 and AC-1 are considerably stiffer, requiring increased stress to reach the same deformation as the rest of the hydrogels, the stress–strain curves registered for the other two composites are similar to the ones registered for the neat hydrogel. This behavior indicates that the minimum ratio of CNTs that has a significant impact on the mechanical properties of the AC hydrogels is 0.5% (wt.) CNT:AAm.

In the case of the PAC series, the highest hysteresis was registered for the neat hydrogel, while the presence of CNTs lowered the hysteresis for all composite materials. The behavior under repeated compressions is also very similar for all compositions; slight differences may be observed only for PAC-1, indicating that the reinforcing effect of the nanoparticles is hindered by the presence of the linear polymer. Compared to the AC series, in which the water around the polymers chains plays the role of lubricant [58] and helps the chains to adjust their position under stress, in the PAC compositions, the linear PAAm also contributes to the polymer chains’ rearrangement during compression, resulting in smaller relaxation times and therefore smaller hysteresis. Continuing the compression cycles leads to an increased hysteresis (the values of the 1st and 10th cycles are presented in Table 3). We attribute the hysteresis cycle to the relaxation time of the materials: a better dispersion of CNTs due to the presence in the initial system of PAAm leads to stiffer materials, as shown by both a reduced hysteresis and a higher breaking stress. However, there is some accumulation of irreversible deformation, as shown by the increase in hysteresis from the 1st to the 10th cycle, but also by the lower stress needed to achieve the same deformation in the 10th cycle as compared with the first (Supplementary Figure S1). For both series, the smallest differences in terms of hysteresis values were registered for the samples with the highest CNTs content: in the case of AC-0, the hysteresis increases from the first to the last compression test with only 0.2%, while in the case of PAC-1, the hysteresis increases with 3.65%.

It is important that the CNTs are homogenously distributed in the polymeric matrix. This is one of the main challenges when working with nanoparticles, and it is very hard to accomplish in low viscosity media [27], such as the precursor in the AC series. The presence of the PAAm in the initial reaction system leads to a significant increase in the viscosity, thus reducing the sedimentation/aggregation tendency of the CNTs to levels low enough to allow the polymerization/cross-linking to be completed before non-homogeneity occurs.

The ability of the materials to withstand changes in length when subjected to compressive loads was assessed through uniaxial compression tests performed at a test speed of 1 mm/s. The ultimate compression stress is considered the stress value reached by the material when it fails completely under the applied load. As presented in Table 3, compositions AC-0.125 and AC-0.25 break at considerably lower stress values compared to the neat hydrogel or the two other composite materials of the same series. This inability to withstand deformation is attributed to the poor dispersion of the nanoparticles. Although agglomerations can also be noticed in AC-0.5, the CNTs seem to have a reinforcing effect on the hydrogel, thus leading to slightly higher values of the ultimate compression stress compared to the neat composition. In this series, the only composition that exhibits a significantly higher resistance to stress compared to the control sample (AC-0) is the one with the highest filling ratio of nanoparticles (AC-1). The addition of the linear polymer (series PAC) leads to a considerably different behavior when compressed to failure. The samples with no or small amounts of CNTs (PAM-0, PAM-0.125, and PAM-0.25) reach a deformation of 85% without breaking, confirming the plasticizer effect of the linear polymer embedded in the 3D network. At higher filling ratios (PAC-0.5 and PAC-1), the effect of the nanoparticle reinforcing becomes visible, and although the materials can withstand higher deformations compared to the other compositions of the series (around 80% deformation), they break when high stress is applied (almost 450 kPa for PAC-0.5 and 630 kPa for PAC-1). The stress–strain curves are presented in Supplementary Figure S2. The compression modulus of the samples was also computed from the stress–strain curves at a deformation of 2%. The results (Table 3) indicate that at small deformations, the addition of nanoparticles does not have a significant impact on the samples’ elasticity. Numerous studies present the possibility to tune the mechanical properties of composites using various CNTs loading ratios [59,60,61,62,63]. For example, in the system MWCNT-poly(vinyl alcohol) (PVA), the addition of 3% wt. CNTs led to an increase in Young’s modulus with about 250% when compared to the neat PVA hydrogel [63]. In a similar CNTs-PAAm system, the addition of 5% wt. carboxyl-functionalized MWCNT increases Young’s modulus 3-fold when compared to a 1% wt. loading [60]. However, there are also studies that present the changes in the composites’ mechanical properties at the addition of low filling ratios. Lan et al. [41] showed that the addition of 0.25% wt. CNT in PVA hydrogel leads to approximately 4-fold increase of the elasticity modulus.

Our study indicates that in the AC series, the addition of small amounts of CNTs (0.125 and 0.25% wt. with respect to AAm) leads to the stiffest materials, but this behavior can be attributed to the poor dispersibility of the nanoparticles in the hydrogel precursor. At 1% (wt.) CNTs:AAm (AC-1), the elasticity modulus increases with about 10% compared to the neat hydrogel. The same increase may be observed for samples PAC-0.5 and PAC-1 compared to the PAC control sample (PAC-0). The higher values of E’ indicate that the addition of PAAm in the classical PAAm-MBA system leads to more elastic materials.

### 3.5. Stability in Simulated Physiological Conditions

**Figure 6:**
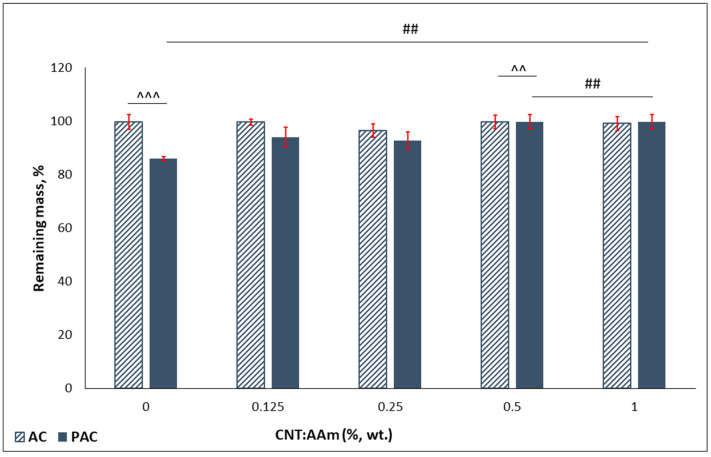
Hydrogels stability after 28 days incubation in PBS. Statistical significance: ^^, ## *p* < 0.01, ^^^ *p* < 0.001.

The stability of purified samples was investigated following incubation for 28 days in PBS, at 37 °C. The remaining mass (RM, %) was computed, and the results are depicted in Figure 6 as bar charts. The AC series is highly stable, with no mass loss. Figure 6 indicates that the presence of linear PAAm reduces the stability of the purified scaffolds, with the strongest effect (statistically significant) recorded for the sample without nanoparticles (PAC-0 registered the highest mass loss, around 14%). The lower values of the RM for the PAC series may be attributed to the solubilization of the linear polymer, immobilized in the 3D polymeric network. The lower values of mass loss (below 10%) recorded for the CNTs-loaded samples indicate that the presence of the nanospecies stabilizes the compositions.

### 3.6. The Antimicrobial Activity

Considering the previously described results indicating that the addition of PAAm to the classical AAm-MBA precursor system leads not only to a visibly improved dispersion of the CNTs but also to more resilient materials to repeated compressions, only the PAC series was subjected to anti-microbial tests.

Due to the well-known antibacterial spectrum of Ag [46], great attention has been paid to the use of Ag-decorated CNTs as efficient antimicrobial agents, but most studies concentrate on the laborious and time-consuming preparation methods, such as the Tollens process [45] or wet impregnation followed by thermal treatment [64]. As the antimicrobial performance of the materials highly depends on the Ag content, several studies discuss the minimum loading of Ag on the CNTs surface. Seo et al. reported that a minimum of 30 µg/mL Ag-MWCNT with a Ag:MWCNT ratio of 2:1 balances both the adequate antibacterial effect and the slightest cytotoxicity when placed in direct contact with both bacteria and cells [65]. The study by Hamouda et al. concludes that 6% represents an optimal Ag loading on the MWCNT surface for efficient bactericidal performances [64]. The antibacterial mechanism of the silver ions has been largely discussed in the reported literature; briefly, the ability of Ag to kill or disrupt the activity of various bacteria is based on the generation of reactive oxygen species, which leads to the membrane disruption [66,67]. Another very important issue to consider is represented by the appropriate amount of silver that would exhibit a relevant antimicrobial effect while being cytotoxic, which differs depending on the application. According to Poon et al., concentrations of silver nitrate higher than 33.3 × 10^−4^% are toxic towards monocultured fibroblasts, while concentrations higher than 60 × 10^−4^% are needed to exhibit toxic behavior towards cells cultured in three-dimensional collagen gel [68]. Khansa et al. recently reviewed the use of silver in wound therapy and concluded that for silver-loaded wound dressing to be both effective and safe, it should have a concentration of silver in the range of 30–60 ppm [69]. Silver has also been employed in various concentrations in nanostructuring composite systems. Three-dimensionally printed bioceramic scaffolds modified with silver-decorated graphite oxide have shown, in addition to osteogenic activity, excellent antimicrobial behavior at concentrations over 3.5 ppm [70].

**Figure 7:**
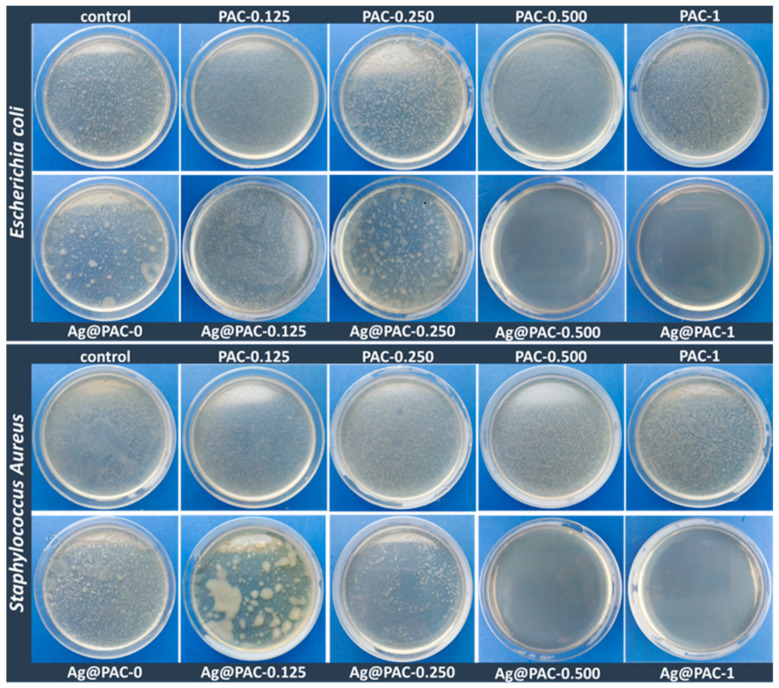
Antimicrobial efficiency of the Ag@PAC series compared to PAC counterparts.

The antimicrobial activity of the PAC materials was assessed using silver decorated CNTs (Ag@CNTs) as fillers, while maintaining the synthesis protocol, as previously described. The ratio Ag:CNT was maintained 1:100 in all samples, resulting in a maximum of 78.4 × 10^−4^% wt./wt. in PAC-1 and half that amount in PAC-0.5. The registered results (Figure 7 and Table 4) show an efficiency of over 95% for Ag@PAC-0.5 and Ag@PAC-1 when tested against the Gram-negative bacteria (*E. coli*). Better results were obtained in the case of Gram-positive bacteria (*S. aureus*), where a Ag@CNT loading ratio of only 0.25% (wt.) with respect to AAm was sufficient for a 50% efficiency. Higher Ag@CNT loading led to higher antimicrobial efficiencies of 98% (for Ag@PAC-0.5) and 100% (for Ag@PAC-1).

## 4. Conclusions

The main challenge in using hydrogels in applications related to tissue regeneration is represented by their inadequate mechanical properties; although hydrogels are usually soft, most of them are not able to withstand high values of stress. The present paper aims to design nanocomposites that exhibit both elasticity and toughness by simultaneously using two different approaches: (1) the embedding of the linear PAAm in the 3D network of the corresponding monomer and cross-linker, aiming to improve CNTs’ dispersion in the precursor and scaffolds’ elasticity, and (2) the use of low ratios of nanoparticles as fillers, with the aim of providing toughness to the so obtained nanostructured system. The addition of the linear polymer (PAAm) in the classical PAAm-MBA system has a great contribution to the dispersibility of the CNTs in the precursor, as it can be easily observed even from the synthesis stage (Figure 1). Our results suggest that adding linear PAAm might contribute to chains’ rearrangement and increase their resistance under load. Furthermore, the PAC materials have greater resistance to compression compared to their AC counterparts. At a low CNT-filling ratio (less than 0.5% CNT:AAm, wt.), the ultimate compression stress could not be reached, while the values registered for PAC-0.5 and PAC-1 were considerably higher when compared to AC-0.5 and AC-1, respectively. This behavior confirms that the addition of the linear PAAm leads to more resistant materials under load, while the nanoparticles reinforce the materials, leading to stiffer compositions. As indicated by micro-computed tomography, there are also some differences with regard to the architectural features of the lyophilized samples. The PAC series exhibit a slightly lower total porosity, and the pores’ dimensions were mostly smaller compared to the AC series. The porosity of both series shows that these compositions are suitable for applications in the field of articular cartilage regeneration and repair. In addition, the paper demonstrates that the decoration of CNTs with low amounts of Ag leads to materials with excellent antimicrobial properties. Our results indicate that these materials have an efficiency of over 95% for both Gram-negative (*Escherichia coli*) and Gram-positive (*Staphylococcus aureus*) bacteria at a Ag loading of only 39.2 × 10^−4^% (wt./wt.) in the final precursor dispersion. The silver content in Ag@PAC-0.5 is within the safety range established by Khansa et al. [69], while in Ag@PAC-1, the silver content is a bit higher. Our findings suggest that the obtained silver-decorated nanocomposites represent effective materials with enhanced mechanical properties and excellent antimicrobial activity which may find potential applications, particularly tissue regeneration and repair.

## Figures and Tables

**Table 1:** Synthesized materials’ denomination and composition of the polymerization mixture.

Series	Composition *	AAm:PAAm, wt./wt.	CNT:AAm, wt./wt.	CNT:Ag, wt./wt.
AC	AC-0	-	0:100	-
	AC-0.125	-	0.125:100	-
	AC-0.25	-	0.25:100	-
	AC-0.5	-	0.5:100	-
	AC-1	-	1:100	-
PAC	PAC-0	100:25	0:100	
	PAC-0.125		0.125:100	-
	PAC-0.25		0.25:100	-
	PAC-0.5		0.5:100	-
	PAC-1		1:100	-
Ag@PAC	Ag@PAC-0	100:25	0:100	100:1
	Ag@PAC-0.125		0.125:100	
	Ag@PAC-0.25		0.25:100	
	Ag@PAC-0.5		0.5:100	
	Ag@PAC-1		1:100	

* Constant parameters: AAm concentration—10% wt./vol. in the final solution; AAm:MBA = 100:2 wt./wt.

**Table 2:** Morphometric parameters registered through micro-CT.

Sample	Specific Surface Area, mm^−1^	Total Porosity, %	Open Porosity, %	Closed Porosity, %
AC-0	437.10	92.584	92.584	0.000
AC-0.125	289.33	91.985	91.983	0.020
AC-0.25	456.90	92.818	92.818	0.000
AC-0.5	384.21	94.607	94.607	0.000
AC-1	310.30	91.438	91.440	0.024
PAC-0	335.98	89.112	89.109	0.027
PAC-0.125	345.72	89.094	89.094	0.000
PAC-0.25	382.09	86.189	86.188	0.002
PAC-0.5	441.45	88.894	88.894	0.000
PAC-1	384.99	91.782	91.782	0.005

**Table 3:** Mechanical parameters of the AC and PAC hydrogels.

Sample	Hysteresis, % 1st Compression Cycle	Hysteresis, % 10th Compression Cycle	Ultimate Compression Stress, kPa	E′ at 2% Deformation, kPa
AC-0	11.55	12.15	212.83 ± 63.08	46.81 ± 3.44
AC-0.125	8.42	9.49	142.49 ± 70.6	54.37 ± 8.00
AC-0.25	8.62	10.06	150.32 ± 12.75	51.10 ± 4.15
AC-0.5	14.76	17.49	285.66 ± 73.4	50.66 ± 5.60
AC-1	12.66	12.68	357.53 ± 26.94	52.31 ± 5.00
PAC-0	11.37	13.25	*	38.95 ± 2.79
PAC-0.125	5.95	6.97	*	39.63 ± 6.17
PAC-0.25	9.36	9.80	*	39.68 ± 2.96
PAC-0.5	6.71	8.66	447.67 ± 75.97	42.44 ± 3.00
PAC-1	7.37	7.65	630.81 ± 88.65	42.93 ± 0.69

* no breakage within the equipment compression limits.

**Table 4:** Silver content (% wt./wt. with respect to the total solid content) and antibacterial efficiency of the PAC and Ag@PAC series against Gram-negative (*E. coli*) and Gram-positive (*S. aureus*) samples.

Sample	Silver Content, % wt./wt.	Antibacterial Activity, %	
		*E. coli*	*S. aureus*
control	-	5	5
PAC-0.125	-	5	5
PAC-0.25	-	15	5
PAC-0.5	-	10	5
PAC-1	-	10	10
Ag@PAC-0	0	25	15
Ag@PAC-0.125	9.8 × 10^−4^	25	25
Ag@PAC-0.25	19.6 × 10^−4^	35	50
Ag@PAC-0.5	39.2 × 10^−4^	95	98
Ag@PAC-1	78.4 × 10^−4^	99	100

**Figure A1:**
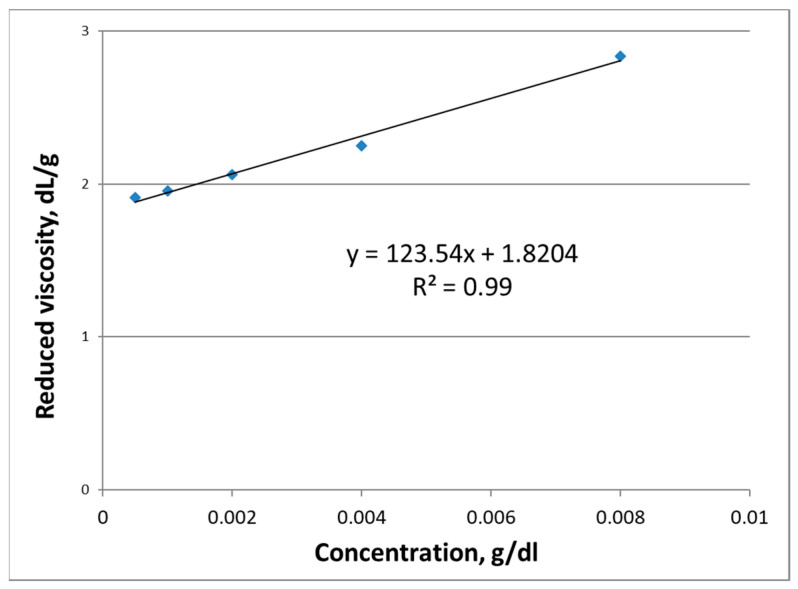
Reduced viscosity of the PAAm aqueous solutions as function of concentration.

## Data Availability

The data presented in this study are available on request from the corresponding author.

## Supplementary Materials

The following supporting information can be downloaded at https://www.mdpi.com/article/10.3390/polym14122320/s1. Supplementary Figure S1: Hysteresis curves for all hydrogels; Supplementary Figure S2: strain–stress curves of the AC and PAC series, respectively; Supplementary Figure S3: Relevant SEM images of the PAC freeze-dried samples, indicating the smooth surface of the pores’ walls.

## Appendix A

Appendix A contains experimental details regarding the determination of the average molecular weight of the synthesized polyacrylamide used in obtaining the PAC and Ag@PAC series.

### Determination of the Average Molecular Weight of the Synthesized Polyacrylamide (PAAm)

An Ubbelohde capillary viscosimeter was employed to determine the relative, specific and reduced viscosity of the synthesized PAAm, using the protocol described in [71]. Based on these data, the average molecular weight was computed, using Mark-Houwink equation (Equation (A1)), expressed as

(A1) $$[\eta ]=K\;\cdot \;M_{V}^{\alpha }$$

where η represents the intrinsic viscosity [dL/g]

*K* and α constant parameters for polymer-solvent solutions (k = 6.31 × 10^−5^ dL/g; α = 0.8 for polyacrylamide-water, at 30 °C [72]).

*M_w_* represents the average molecular weight [Da].

Briefly, several PAAm aqueous solutions were prepared, with concentrations ranging from 0.05 g/dL to 0.8 g/dL were prepared. The solutions and the solvent were filtered and subsequently introduced in the Ubbelohde viscosimeter, placed in a water bath with a precise control of the temperature, at 30 °C. The flowing time of each solution is measured, and the average is used to determine the relative viscosity following Equation (A2).

(A2) $$\eta_{rel}=\frac{\tau_{solution}}{\tau_{solvent}}$$

(A3) $$\eta_{sp}=\eta_{rel}-1$$

To obtain the intrinsic viscosity a graph $\frac{\eta_{sp}}{c}=f\left(c\right)$ was plotted (Figure A1) and the intrinsic viscosity was read by extrapolating the concentration to zero.

By substituting the obtained value for the intrinsic viscosity (1.8204 dL/g) in Mark-Houwink equation (Equation (A1)), the obtained average molecular weight of the synthesized PAAm is 3.76 × 10^5^ Da.
