# Supplementary material for: Nanostructured Polyacrylamide Hydrogels with Improved Mechanical Properties and Antimicrobial Behavior
Source: Polymers (Basel). 2022 Jun 8;14(12):2320. doi: 10.3390/polym14122320 (PMC9227893; doi:10.3390/polym14122320)
Supplement: Supplementary file 1 [file polymers-14-02320-s001.zip › Supplementary figure S1.pdf]

Supplementary figure S1: Hysteresis curves for all hydrogels

Hysteresis curves for the AC series. First and last cycles are highlighted in back and red, respctively.

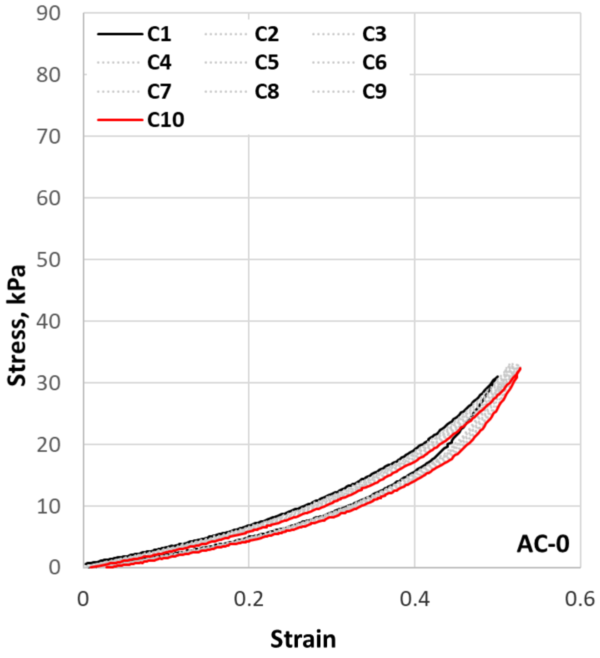

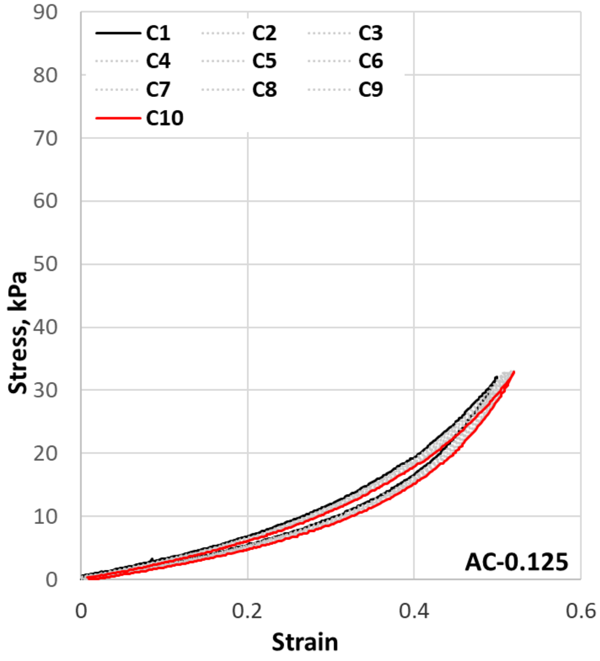

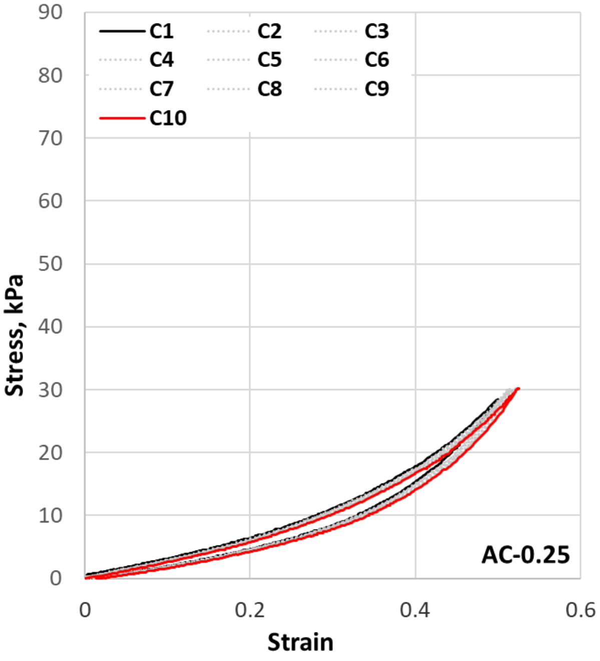

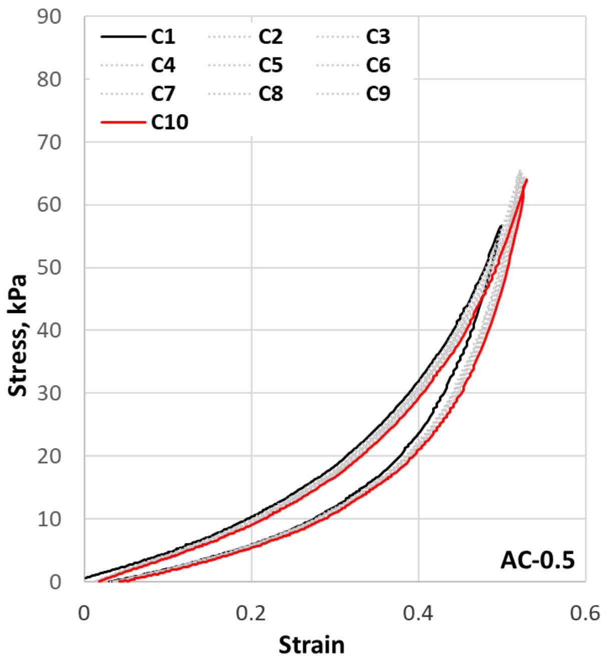

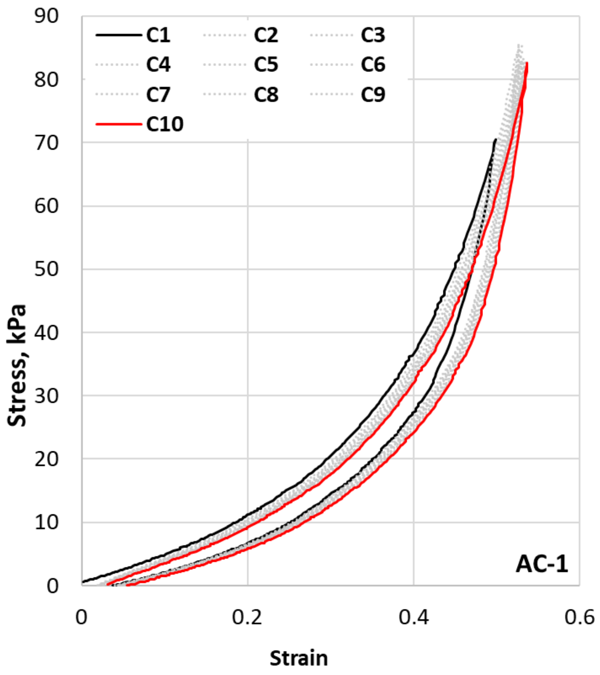

Hysteresis curves for the PAC series. First and last cycles are highlighted in back and red, respctively.

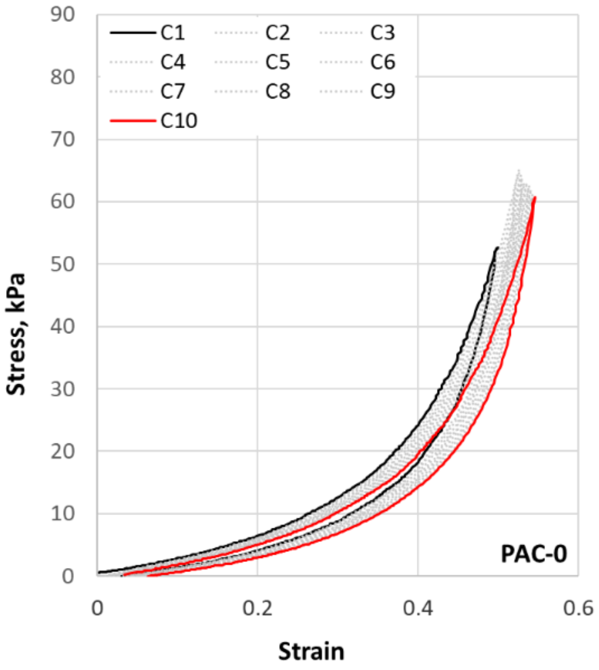

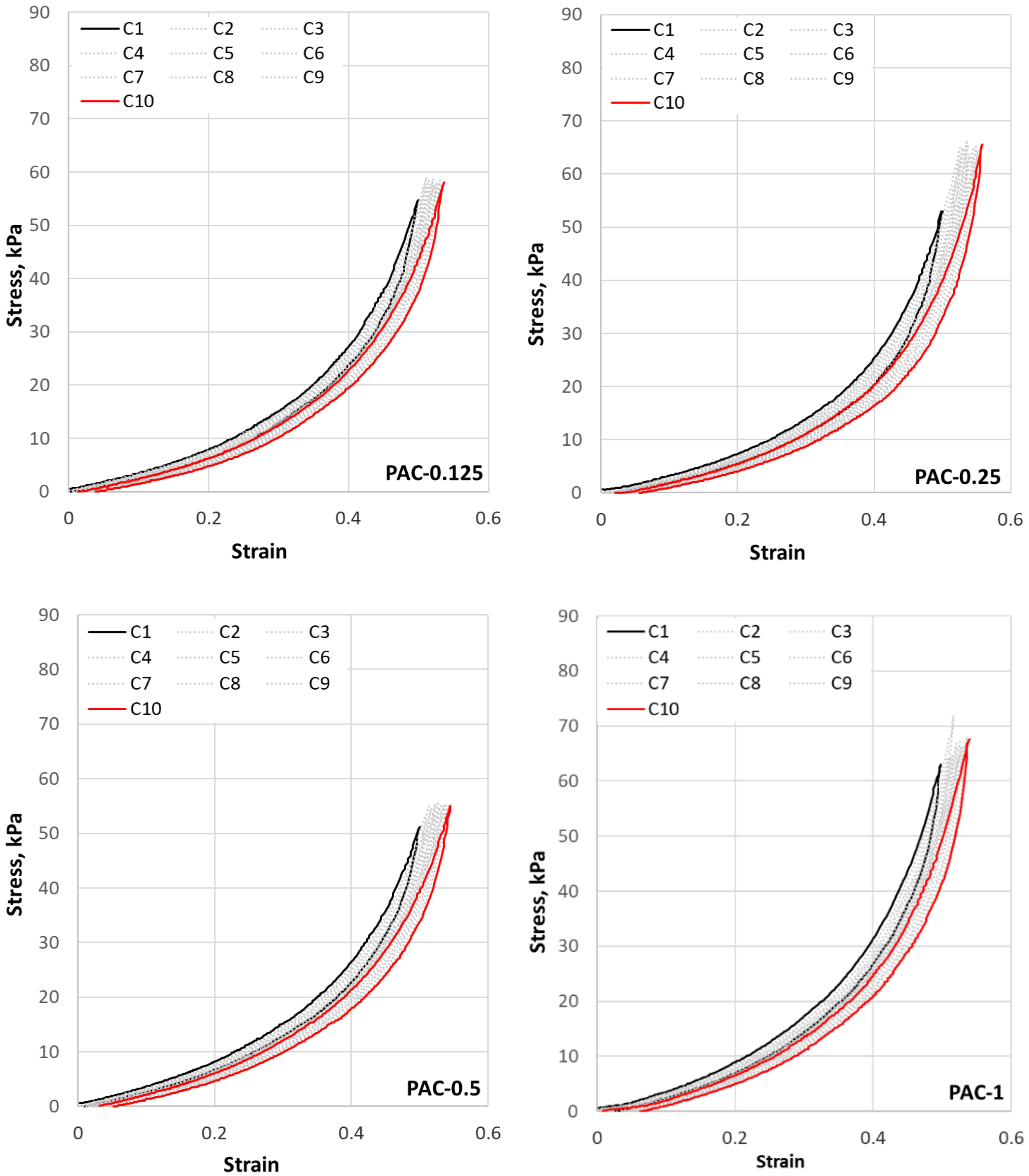
Stress, kPa
Strain
C1
C2
C3
C4
C5
C6
C7
C8
C9
C10
PAC-0.125
PAC-0.25
PAC-0.5
PAC-1
