# Supplementary material for: Nanostructured Polyacrylamide Hydrogels with Improved Mechanical Properties and Antimicrobial Behavior
Source: Polymers (Basel). 2022 Jun 8;14(12):2320. doi: 10.3390/polym14122320 (PMC9227893; doi:10.3390/polym14122320)

Supplementary figure S3: Relevant SEM images of the PAC freeze-dried samples, indicating the smooth surface of the pores' walls.

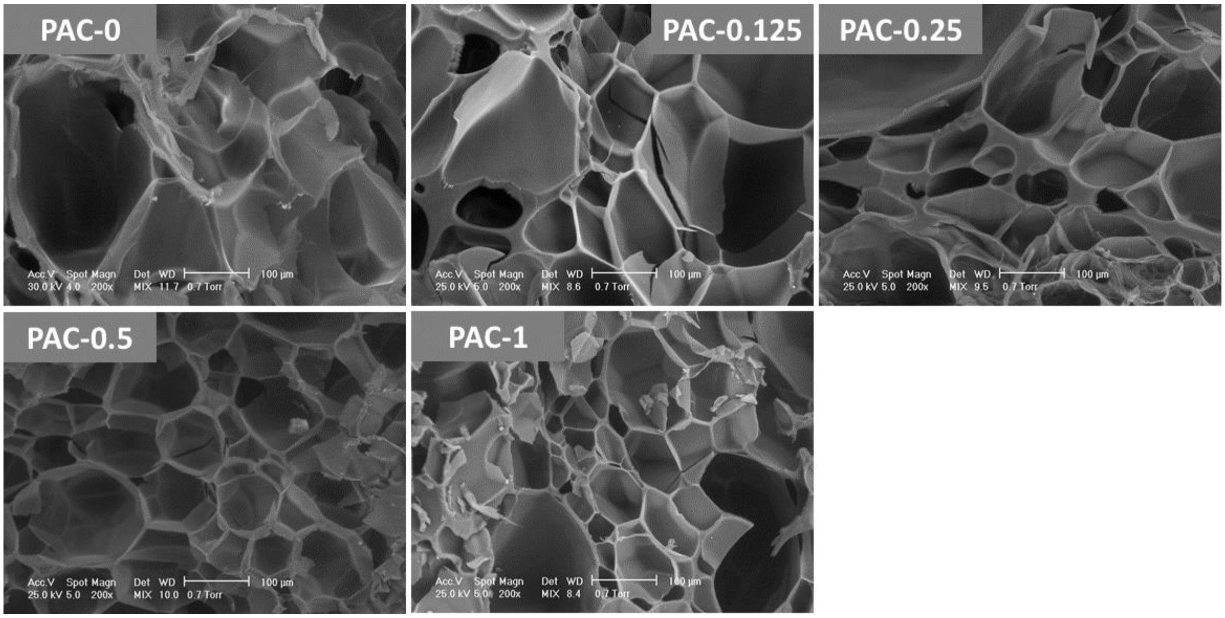

Supplement: Supplementary file 1 [file polymers-14-02320-s001.zip › Supplementary figure S3.pdf]
